# 

**DOI:** 10.1192/bjb.2023.10

**Published:** 2023-12

**Authors:** Matthew Turner

**Affiliations:** is Consultant Addictions Psychiatrist, Substance Use Service, NHS Forth Valley, Stirling, UK. Email: matthew.turner@nhs.scot

**Figure:**
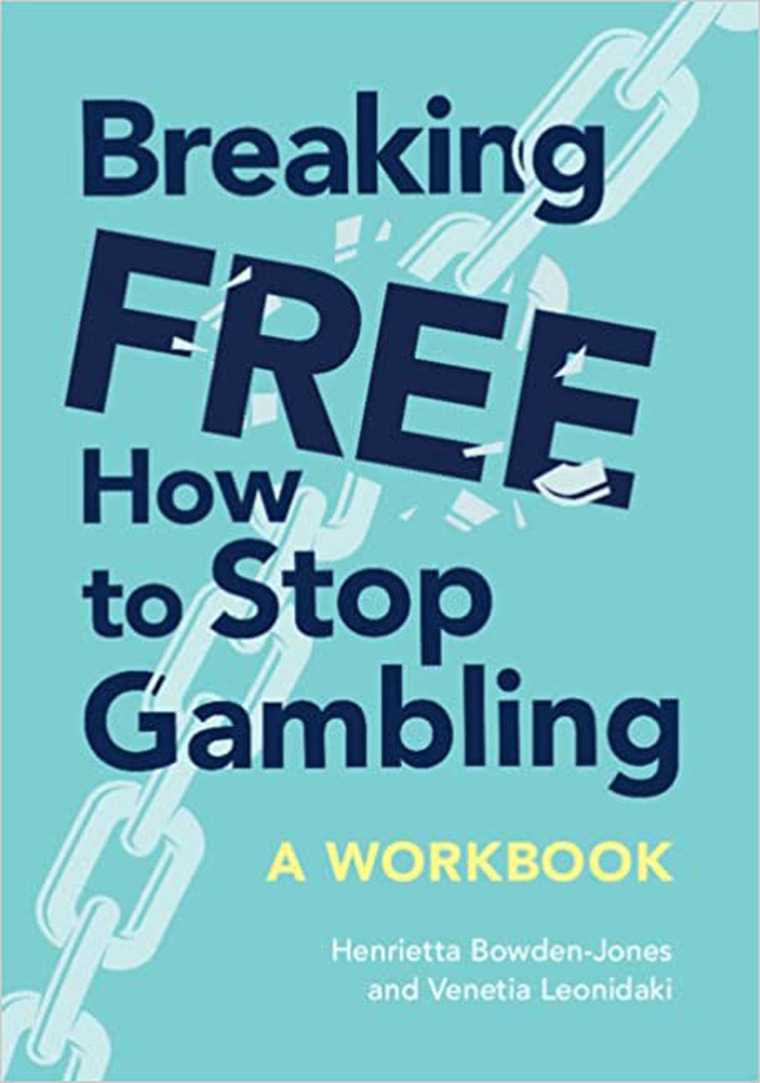

*Breaking Free: How to Stop Gambling* is a very welcome addition to the literature on gambling addiction. In recent years there has been an increasing awareness of the damage done by gambling addiction at both individual and societal levels. A YouGov prevalence study in 2020 estimated that up to 2.7% of adults in Great Britain (1.4 million people) were problem gamblers.

This is a self-help workbook aimed at the individuals affected by gambling-related harms and their significant others. It also serves as an excellent summary of the topic for clinicians. It is co-edited by Professor Henrietta Bowden-Jones, who set up the first NHS clinic for gambling disorders in 2008 and is now the national clinical adviser for gambling harms, and Dr Venetia Leonidaki, a consultant clinical psychologist at the National Problem Gambling Clinic, London.

The book is organised into 15 chapters, which cover topics including how to recognise gambling addiction, the science and physiology underpinning gambling addiction, and strategies to address gambling disorder. The chapter on recognising gambling disorder lays out the Problem Gambling Severity Index and the DSM-5 criteria for gambling disorder. The treatment chapters are based on the cognitive–behavioural therapy protocol used at the National Problem Gambling Clinic. This draws on the evidence-based approach developed by clinical psychologist Dr Nancy Petry in the late 1990s to early 2000s in the USA, focusing on ‘cognitive restructuring’. There is a focus on relapse prevention, including an acceptance that lapses are part of the journey. The book's approach also draws on motivational enhancement therapy, meta-cognitive techniques and mindfulness.

The book is well laid out, with a mixture of practical exercises and activity sheets. Reflecting the widespread use of the internet and mobile phones, there is an accompanying website where resources can be downloaded, including activity sheets. There is also a section recommending apps to download to mobile phones and tablets to track progress.

Each chapter includes case studies and anecdotes that help to illustrate the topic and make it more accessible. At the end of each chapter the most important points are summarised. There is also a useful appendix at the end of the book focused on when to consider medication (naltrexone) in treating gambling disorder. Reflecting the needs of its target audience, it is written in a clear style that avoids unnecessary jargon but provides enough depth to make it useful for both clinicians and those without medical training.

